# Open reduction and internal fixation combined with hinged elbow fixator in capitellum and trochlea fractures: A retrospective study of 15 patients followed for 29 months

**DOI:** 10.3109/17453671003685475

**Published:** 2010-04-06

**Authors:** Giuseppe Giannicola, Federico M Sacchetti, Alessandro Greco, Giuseppe Gregori, Franco Postacchini

**Affiliations:** Department of Orthopaedic Surgery, University “Sapienza” of Rome, RomeItaly

## Abstract

**Background and purpose** The current surgical treatment for displaced fracture of the capitellum and trochlea is open reduction and internal fixation (ORIF), but the results are often unsatisfactory, particularly with complex fractures. Furthermore, the surgical approach, the kind of osteosynthesis, and postoperative management are controversial. We evaluated the results of internal fixation combined with hinged external fixation.

**Methods** We analyzed 15 patients with a mean age of 47 (18–65) years. Based on the Bryan-Morrey-McKee classification, the fractures were identified as type I in 6 cases and type IV in 9. Active and passive motion was started and activities of daily living were permitted on the second postoperative day. The mean follow-up time was 29 (12–49) months.

**Results** In 13 cases, functional range of motion was obtained within 6 weeks of surgery. At final follow-up, 14 patients had a stable, pain-free elbow with a mean active range of motion of 13° to 140°. The average score on the Mayo elbow performance score was 98.

**Interpretation** The use of the hinged fixator allows early motion of the elbow while preserving joint stability. It may have additional value in complex articular fractures when stable internal fixation cannot be obtained with ORIF, and in the presence of severe ligamentous injuries.

## Introduction

Coronal shear fractures of the articular surface of the distal humerus involve the humeral trochlea and/or the capitellum. Capitellar fractures account for only 1% of all elbow fractures and 6% of all distal humeral fractures (Bryan and Morrey 1985, Harrington and McKee 2000), whereas isolated trochlear fractures have only occasionally been observed (Nakatani et al. 2005). All these injuries are frequently unrecognized, and most surgeons have limited experience in their treatment. The short-term treatment failures are joint stiffness and instability, whereas the long-term complication is posttraumatic osteoarthritis of the elbow.

Many treatments have been advocated for these injuries, including closed reduction (Dushuttle et al. 1985, Ochner et al 1996), open reduction and internal fixation (Ring et al. 2003, Dubberly et al. 2006, Ruchelsman et al. 2008), excision of the fracture fragments (Collert 1977, Grantham et al. 1981), prosthetic replacement (Jakobsson 1957, Cobb and Morrey 1997), and fixation or excision of the fragments under arthroscopy (Hardy et al. 2002). In open reduction and internal fixation the surgical approach, the type of osteosynthesis, and the postoperative management are controversial, and the results published have been discordant (Mc Kee et al. 1996, Ring et al. 2003, Dubberly et al. 2006).

Hinged external fixation has been used as a modality of treatment in various elbow conditions, e.g. in managing complicated fracture dislocations and also joint instability after extensive contracture release (Tan et al. 2005, Yu et al. 2007). We believed that articular distraction and neutralization of compression and shear forces by the fixator might also protect open reduction and internal fixation (ORIF) performed for articular fractures and favor ligament healing, thus allowing early postoperative motion of the elbow. We used a hinged external fixator after ORIF in displaced capitellar fractures that were either associated with or not associated with trochlear fractures. We evaluated the results of this treatment in terms of recovery of full range of motion and stability of the elbow.

## Patients and methods

Between January 2003 and July 2006, 15 patients (10 women) with isolated capitellar fracture, or both capitellar fracture and trochlear fracture, underwent surgical treatment. The mean time interval from injury to operation was 5 (2–8) days in 14 cases, while in 1 patient (case 2) surgery was performed 9 weeks after trauma. In this patient, who was seen by us because of persistent pain and stiffness of the elbow, the fracture had not been recognized in another hospital.

The mean age at operation was 47 (18–65) years. All fractures were closed and had occurred following a fall onto the elbow or the outstretched hand, or in motor vehicle accidents. Results of neurovascular examination were normal. Patients underwent plain radiographs and CT scan with 3-D reconstructions. Associated injuries included posterior elbow dislocation in 4 patients (all of whom had lesion of both collateral ligaments (LCL and MCL)), fracture of the lateral epicondyle in 3, fracture of the medial epicondyle in 1, and lesion of the medial collateral ligament (MCL) in 5. Capitellar and trochlear fractures associated with unicondylar, bicondylar, or intercondylar fractures of the distal humerus were not included in the study.

Preoperatively, based on radiological findings, 6 type-I and 9 type-IV fractures according to the Bryan-Morrey (1985) and McKee (1996) classification were identified. The fractures were also classified based on the method of Dubberly et al. (2006), and 3 type-IA, 3 type-IB, 5 type-IIA, 2 type-IIIA, and 2 type-IIIB fractures were identified. Classification of the fractures was performed independently by 2 examiners. In cases of disagreement, a third examiner classified the fracture; it was then allocated to the subgroup chosen by the majority of the examiners. Although our study was retrospective, the diagnostic and therapeutic protocol and the preoperative and postoperative evaluation of patients were similar in all cases.

### Surgical technique

A single surgeon performed all the operations. The patient was placed supine on the operating table with the arm supported on a hand-table. Under general and/or axillary block anesthesia, varus-valgus stress tests—flexion and extension in varying degrees of forearm rotation—were performed under fluoroscopy to detect any instability of the elbow. In 12 patients, a lateral Kocher approach was carried out. The exposure was extended proximally and the origin of the wrist and digital extensor muscles was partially detached from the lateral epicondyle. The lateral ulnar collateral ligament (LUCL) was not released. When a satisfactory exposure could not be obtained, the LUCL was detached from its origin on the lateral epicondyle and repaired after fracture fixation. The surgical exposure was simplified in those cases in which the LCL was disrupted. In 3 patients with a fracture extending to the medial trochlea, a posterior midline incision was made. If the medial aspect of the trochlea could not be visualized adequately from the lateral exposure, the deep dissection was made medially, between the flexor-pronator muscles, leaving the flexor carpi ulnaris attached to the humerus.

After capsulotomy, the fracture fragments were identified, repositioned, and fixed provisionally with Kirschner wires. Definitive internal fixation was achieved with 2–6 Herbert screws, inserted over K-wires, depending on the fracture pattern; however, at least 2 screws were used to ensure rotational stability. The epicondyle fragment was re-attached with cancellous bone lag screws and/or Herbert screws in 4 patients. The position and orientation of the screws was checked with fluoroscopy. The common extensor and/or flexor muscles of the wrist and fingers and the LCL were reinserted with anchors and/or transosseous sutures. A hinged external fixator was applied in all patients. The DJD elbow fixator (Stryker) was used in 11 patients and the OptiROM elbow fixator (Biomet) in 4. The fixator was slightly distracted and blocked with the elbow fully extended to reduce bleeding of the anterior vessels. MCL was not specifically addressed surgically.

### Postoperative management

Indometacin (100 mg daily) was administered for 5 weeks to prevent heterotopic ossifications. 2 days after surgery, the external fixator was unblocked and the patient was encouraged to perform active and passive motion of the elbow. Patients were discharged mean 5 (3–8) days after surgery. Activities of daily living with no restriction were permitted from the second postoperative day. The external fixator was removed 6 weeks after operation; at the time of removal under anesthesia, stability of the elbow was tested with caution so as not to risk disruption of the sutures and osteosynthesis. Strengthening exercises were started when radiographs showed evidence of fracture healing, or at most 6 months after surgery.

### Clinical evaluation

Patients were examined every 3 weeks in the first 3 months, every 6 weeks in the subsequent 3 months, and then every 3 months. After 1 year, the evaluation was done every 12 months. Final follow-up was carried out at an average of 29 (12–49) months. At each follow-up, patients were evaluated using the Mayo elbow performance score (MEPS) and radiographs of the elbow were carried out. Loss of muscle strength of arm and forearm was evaluated according to the classification of the British Medical Research Council (MRC), which grades muscle strength on a scale from 0 (paralysis) to 5 (normal strength) (Seddon 1972).

### Statistical analysis

Examiners' agreement on imaging classification of the type of fracture (by radiographs and CT) was determined using the Cohen's kappa-index of inter-rater reliability.

## Results

**Figure 1.**
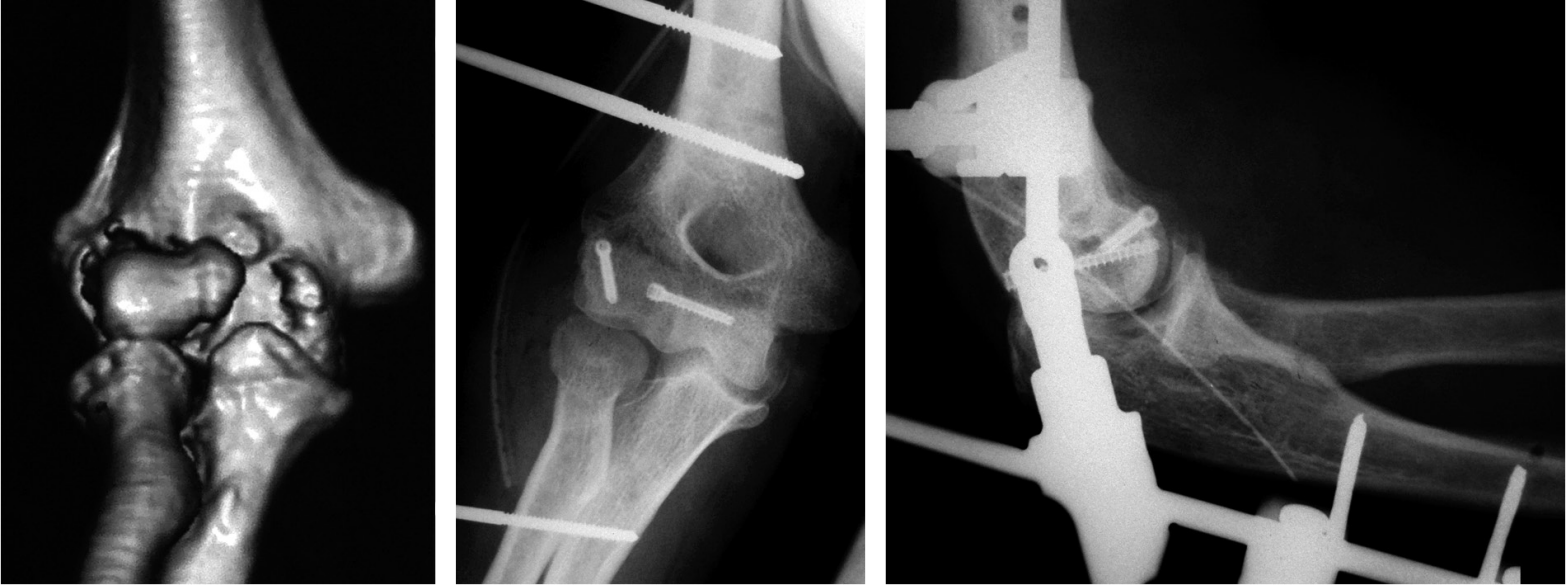
Case 1. 3-D CT reconstruction showing displaced fracture of the capitulum humeri and trochlea. Internal fixation screws supplemented with a hinged external fixator.

**Figure 2.**
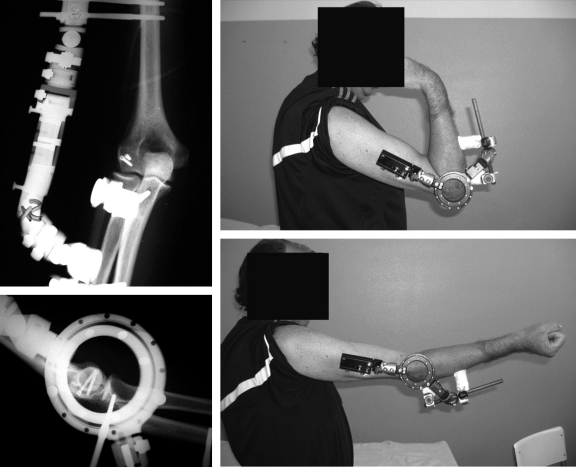
Case 4. Postoperative radiographs showing internal fixation with screws and hinged fixator. Photographs 3 weeks after surgery showing recovery of motion.

13 patients recovered or exceeded the functional range of motion (30°–130° extension–flexion and 50°–50° pronation–supination) in 6 weeks; 1 patient achieved the same functional result in 3 weeks (Table, Figures 1 and 2). Only the patient who had a late operation (case 2) did not recover normal motion. In the other patients, the mean extension was 13° (0–40) and the mean flexion was 140° (110–150) 3 months after surgery. This range of motion did not change until the final follow-up. All patients had full pronation and supination. At final evaluation no patients complained of pain, except for 1 who had moderate discomfort during physical effort. In all cases the elbow was stable, except for 1 patient (case 7) who had moderate instability at the time of removal of the fixator and at the final evaluation. All patients recovered normal muscle strength. All were satisfied with their outcome and all of them had returned to their previous activity levels. The average score on the MEPS was 98 (75–100), corresponding to an excellent outcome. No differences in the quality of outcome were found depending on whether the fractures were classified with the system of Bryan-Morrey (1985) and McKee (1996) or with that of Dubberly et al. (2006).

**Table:** Degrees of elbow extension–flexion (top line) and pronation–supination (bottom line) for each of the 15 patients

Patient no.	Age	Sex	Classification		Range of motion (°)					
			Morrey	Dubberly	3 weeks	6 weeks	9 weeks	3 months	12 months	Final follow-up
1	62	F	IV	IIa	20–120	20–130	10–150	0–150	0–150	0–150
					80–80	80–80	80–80	80–80	80–80	80–80
2	51	F	IV	IIa	40–120	40–100	45–100	40–110	40–110	40–11
					80–85	80–85	80–85	80–85	80–85	80–85
3	38	F	IV	IIa	10–120	10–140	10–140	0–140	0–140	0–140
					60–60	70–85	75–80	75–80	75–80	75–80
4	47	M	I	Ia	25–130	10–130	10–140	0–150	0–150	0–150
					85–90	85–90	85–90	85–90	85–90	85–90
5	60	F	I	Ib	40–120	30–135	30–140	35–140	35–140	35–140
					40–80	60–80	70–80	35–140	35–140	80–90
6	38	F	IV	IIIa	40–100	25–135	20–135	20–140	20–140	20–140
					80–65	80–70	80–75	85–70	85–70	85–70
7	55	F	IV	IIa	40–120	30–135	30–140	25–150	25–150	25–150
					80–80	80–80	80–80	80–80	80–80	80–80
8	30	M	IV	IIIb	40–100	25–130	30–135	20–140	20–140	20–140
					60–50	75–75	80–80	80–80	80–80	80–80
9	18	M	IV	IIa	40–115	20–135	10–145	10–150	10–150	10–150
					70–40	70–80	80–80	80–80	80–80	80–80
10	57	M	I	Ib	20–130	15–135	5–130	10–135	5–135	5–135
					80–50	80–65	80–80	80–80	80–80	80–80
11	57	F	IV	III a	30–100	25–140	20–135	10–140	10–140	10–140
					60–70	60–70	55–80	70–80	80–80	80–80
12	26	M	I	Ia	5–90	15–140	5–135	10–145	10–145	10–145
					80–80	80–80	80–80	80–80	80–80	80–80
13	65	F	IV	IIIb	45–90	25–130	15–130	5–135	5–135	5–135
					75–55	80–70	80–80	80–80	80–80	80–80
14	41	F	I	Ia	30–110	35–135	10–140	5–145	5–145	5–145
					50–60	70–70	80–80	80–80	80–80	80–80
15	59	F	I	Ib	25–110	30–130	20–140	10–140	10–140	10–140
					60–40	70–80	70–80	80–80	80–80	80–80

**Figure 3.**
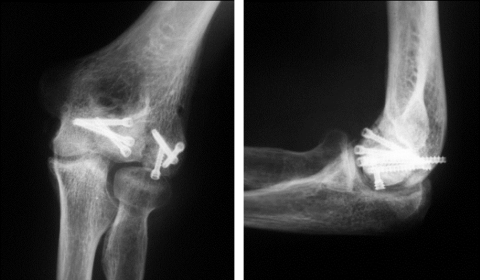
Case 6. Anteroposterior and lateral radiographs taken 6 months after surgery showing minimal bone resorption of the capitellum, moderate lateral compartment osteoarthritis, and partial extrusion of a Herbert screw.

Fracture union occurred in 14 cases, with no evidence of avascular necrosis. In 1 patient (case 7), radiographs obtained at 9-month follow-up showed a pseudarthrosis that was asymptomatic and partial extrusion of a Herbert screw, which was removed. At final follow-up, 2 patients had minimal bone resorption of the capitellum with moderate lateral compartment osteoarthritis, which had no influence on the clinical result. One of these 2 patients (case 6) had a partial extrusion of a Herbert screw, which was removed (Figure 3). No other patients underwent repeat surgery. One patient (case 2) had a postoperative motor deficit of the radial nerve, related to humeral pin placement, which recovered completely 8 months after operation. There was no delay in wound healing or heterotopic ossification. One patient had a superficial pin tract infection, which resolved with local wound care and oral antibiotics. At the final follow-up, 3 patients showed mild osteoarthritic changes. However, they were asymptomatic and both the anatomical reduction of the fracture and the active range of elbow motion were excellent or good.

In the preoperative classification of the fractures on plain radiographs with the system of Bryan-Morrey (1985) and McKee (1996) or that of Dubberly et al. (2006), there was moderate agreement between the observers (κ = 0.54 and 0.50, respectively). Instead, the classification performed on CT scans revealed an almost complete agreement for both classification methods (κ = 1).

## Discussion

Coronal shearing fractures of the distal humerus are uncommon and difficult to identify accurately on plain radiographs. It is essential to obtain a perfect lateral view, in addition to the AP view, because oblique views may not reveal the presence or pattern of the fracture. In 1 patient in our series, the fracture was unrecognized in another hospital and was diagnosed in our hospital 9 weeks after the trauma. Classification based on plain radiographs is also difficult, as shown by the moderate agreement between initial observers in our study. These injuries may initially appear as isolated capitellar fractures, but on CT scans they are often found to extend into the anterolateral trochlea and, in some cases, to involve the posterior aspect of the lateral column, the posterior trochlea, and the epicondyles (Ring et al. 2003). In our study, after CT scanning, 6 fractures were re-classified. We thus believe that CT, with 3-D reconstructions, should always be performed to make a precise diagnosis and allow accurate preoperative planning.

Closed reduction of capitellum and/or trochlear fractures rarely achieves and maintains fragment reduction, and requires a long period of immobilization. A few authors (Alvarez et al. 1975, Grantham et al. 1981) have excised small capitellar fragments, with satisfactory results in the short term. However, resection of capitellar fragments predisposes to capsular adhesions, resulting in restricted mobility of the elbow. Furthermore, it leads to valgus instability of the elbow in the presence of MCL injury, which is often difficult to diagnose in recent fractures (Dushuttle et al. 1975, Grantham et al. 1981, Hendel and Halperin 1982). In 5 of our patients, MCL injury could be diagnosed only under general anesthesia by manipulation under fluoroscopy. Moreover, 4 other patients had a posterior elbow dislocation.

The most common treatment of displaced fractures of the capitulum humeri and/or trochlea is ORIF (Lansinger and Måre 1981, Ring et al. 2003, Dubberly et al. 2006, Ruchelsman et al. 2008), which is intended to restore joint congruity and to allow early rehabilitation. Previous studies (Mc Kee et al. 1996, Ring et al. 2003, Dubberly et al. 2006, Ruchelsman et al. 2008) have suggested that the results of surgery are related to the type of fracture, the kind of osteosynthesis, and the kind of postoperative management. In some of these fractures, internal fixation provides sufficient interfragmentary stability to allow immediate postoperative mobilization with good results (Sano et al. 2005, Ruchelsman et al. 2008). When osteosynthesis does not ensure enough stability to allow early motion, as occurs in most cases, the elbow is immobilized to avoid fragment displacement or fracture nonunion. In these cases, however, the immobilization may lead to elbow stiffness. It is also known that these fractures involve a high rate of reoperation, which in 2 recent series was 10/21 and 12/28 due to elbow contracture, ulnar neuropathy, early loss of fixation, or elbow discomfort requiring hardware removal (Ring et al. 2003, Dubberly et al. 2006). The good results in terms of the recovery of ROM obtained in almost all our patients are most probably related to the very precocious postoperative elbow motion permitted by the external fixator—which slightly distracted the elbow joint, thus protecting ORIF, and stabilized the joint even in most complex injuries. In contrast to Ruchelsman et al. (2008), we found similar outcome in type-I and type-IV fractures (Bryan-Morrey-McKee classification) regarding elbow flexion and extension.

The rate of complications in our patients was similar to, or lower than, that found in recently reported series of capitellar and/or throclear fractures (Ring et al. 2003, Dubberly et al. 2006). However, in contrast to the latter series, only 2 of our patients required reoperation for removal of a loosened screw, with no influence on the functional result. In our series, 2 complications (1 radial neurophaty and 1 pin tract infection) were related to the use of the external fixator. This is similar to the rate reported by Cheung et al. (2008).

In the first 11 patients in our series, the DJD elbow fixator (Stryker) was used, but we experienced difficulties in inserting the axis pin in the center of rotation of the elbow joint. In fact, the anatomical axis of rotation lies at the center of the capitellum and trochlea, and the axis pin can thus intersect the screws used for internal fixation. Hence, in the last 4 cases, we used the OptiROM elbow fixator (Biomet) because it allows the axis of rotation of the elbow to be located without using any pin. Furthermore, this fixator permits the articular congruity to be checked through the axis guide ring after fixator positioning and the healing process of fracture to be evaluated more accurately.

A limitation of our study was the short follow-up. Our results indicate that osteoarthritic changes may develop early on, even in the presence of a good reduction of the fracture, but it is unclear whether they may become symptomatic later on.

In conclusion, the results of this study show that the association of hinged external fixation with ORIF allows a very fast functional recovery of the elbow due to early unrestricted postoperative motion. The use of a hinged external fixator may be indicated in complex articular fractures, particularly when associated with ligamentous injuries, and when a stable fixation of the fracture fragments cannot be obtained with ORIF.
